# Metabolic regulation of forkhead box P3 alternative splicing isoforms and their impact on health and disease

**DOI:** 10.3389/fimmu.2023.1278560

**Published:** 2023-10-06

**Authors:** Zhidan Luo, Yihua Zhang, Qais Waleed Saleh, Jie Zhang, Zhiming Zhu, Martin Tepel

**Affiliations:** ^1^ Department of Geriatrics, Chongqing General Hospital, Chongqing, China; ^2^ Cardiovascular and Renal Research, Department of Molecular Medicine, University of Southern Denmark, Odense, Denmark; ^3^ Department of Cardiology, Chongqing Fifth People’s Hospital, Chongqing, China; ^4^ Department of Nephrology, Odense University Hospital, Odense, Denmark; ^5^ Department of Hypertension and Endocrinology, Daping Hospital, Chongqing, China

**Keywords:** forkhead box P3, splicing isoforms, metabolic regulation, regulatory T cells, suppressive function, glycolysis, fatty acid oxidation, autoimmune diseases

## Abstract

Forkhead Box P3 (FOXP3) is crucial for the development and suppressive function of human regulatory T cells (Tregs). There are two predominant FOXP3 splicing isoforms in healthy humans, the full-length isoform and the isoform lacking exon 2, with different functions and regulation mechanisms. FOXP3 splicing isoforms show distinct abilities in the cofactor interaction and the nuclear translocation, resulting in different effects on the differentiation, cytokine secretion, suppressive function, linage stability, and environmental adaptation of Tregs. The balance of FOXP3 splicing isoforms is related to autoimmune diseases, inflammatory diseases, and cancers. In response to environmental challenges, FOXP3 transcription and splicing can be finely regulated by T cell antigen receptor stimulation, glycolysis, fatty acid oxidation, and reactive oxygen species, with various signaling pathways involved. Strategies targeting energy metabolism and FOXP3 splicing isoforms in Tregs may provide potential new approaches for the treatment of autoimmune diseases, inflammatory diseases, and cancers. In this review, we summarize recent discoveries about the FOXP3 splicing isoforms and address the metabolic regulation and specific functions of FOXP3 splicing isoforms in Tregs.

## 1 Introduction

CD4^+^CD25^+^ regulatory T cells (Tregs) are a subset of T cells that mediate the immune response against antigens and inhibit conventional T-cell activation and proliferation (1, 2). Tregs generally suppress the excessive immune response and maintain immune homeostasis. In contrast, the deficiency or deactivation of Tregs leads to autoimmune diseases or allograft rejection (1, 2).

The phenotype and function of Tregs critically depend on the expression of their lineage-defining master transcription factor Forkhead Box P3 (FOXP3) (3). Unlike in mice, alternative splicing of mRNA leads to 4 different FOXP3 protein isoforms in human Tregs (4). The full-length isoform (FOXP3FL) and a shorter isoform produced by transcripts lacking exon 2 (FOXP3ΔE2) are the predominant isoforms in healthy human natural Tregs (1) and they are the primary focus of this review. Both isoforms have previously been shown to induce differentiation of CD4^+^ T cells to Treg phenotypes, and their relative expression varies in some autoimmune diseases, inflammatory diseases, and cancers (5, 6). The other 2 isoforms, FOXP3 with exon 7 skipping (FOXP3ΔE7) and FOXP3 missing both exon 2 and exon 7 (FOXP3ΔE2ΔE7) have been reported with very low frequency in human natural Tregs (1, 7–11). The isoforms of FOXP3 exhibit differences in functions (12–14) and regulation mechanisms (1, 15, 16).

Metabolic reprogramming in immune cells is essential for their proper function, leading to the process called immunometabolism (17). Tregs can adapt quickly to both intrinsic and extrinsic microenvironments by metabolic reprogramming to maintain their activity (18). FOXP3 seems to be a critical link between the energy metabolism and functions of Tregs. FOXP3 can regulate energy metabolism and control the differentiation, stability, and suppressive function of Tregs (19, 20). Modulation of metabolic pathways can also affect FOXP3 transcription and splicing (16, 17), which may be important for controlling the function of Tregs both in healthy and diseased subjects.

In this review, we summarize recent discoveries about the FOXP3 splicing isoforms, especially about FOXP3FL and FOXP3ΔE2, and address the specific functions and metabolic regulation of these isoforms in Tregs.

## 2 Functions and balance of FOXP3 isoforms

### 2.1 FOXP3 gene and protein structures

**Figure 1:**
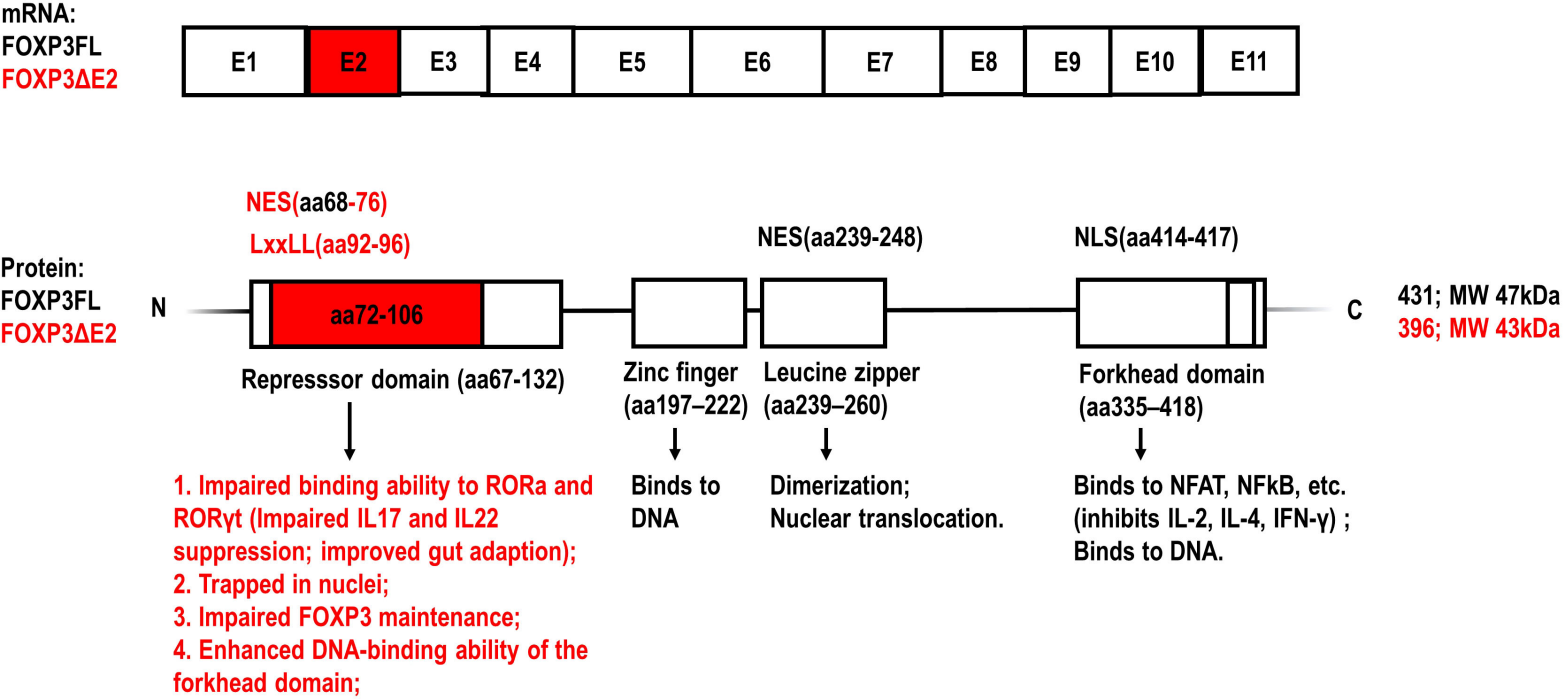
Schematic representation of the exons of *FOXP3* mRNA and the structural domains of FOXP3 protein (1, 11, 21). The amino acid sequences encoded by *FOXP3* exon 2 and the specific functions of FOXP3 isoform lacking exon2 (FOXP3ΔE2) are highlighted with red color, compared to the full-length FOXP3 isoform (FOXP3FL). NES, nuclear export signal; NLS, nuclear localization signal; LxxLL (where L is leucine and x is any amino acid) motif: a multifunctional binding sequence in transcriptional regulation.

*FOXP3* gene is located at Xp.11.23 on the X chromosome and contains 12 exons. The first exon is noncoding while the remaining 11 are coding exons (1, 8). Although some articles name all exons, including the non-coding exon as E1, most articles refer to the coding exons as E1-11 and name the first exon as the non-coding exon, which is also the way *FOXP3* exons are referred to hereafter in this article. The full-length human FOXP3 protein has 431 amino acids with a molecular weight of about 47.25 kDa (1, 8). FOXP3 has 4 main domains with different functions (**Figure 1**). From the N terminus, it contains a repressor domain, a zinc finger, a leucine zipper motif, and finally a forkhead DNA-binding domain (1, 8).

The proline-rich repressor domain exerts a suppressive effect on target genes (22, 23). The function of the zinc-finger domain has not been elucidated clearly yet (22, 23). The leucine zipper motif enables FOXP3 dimerization, which is required for binding to the GTAAACA motif via the C-terminal forkhead domain (22, 23). The forkhead DNA-binding domain regulates the transcription of approximately 700 genes involved in a wide spectrum of inflammatory and immune responses (1). AlphaFold2 predictions and *in vitro* experiments demonstrate that the N-terminal domain including exon 2 of FOXP3 inhibits the DNA binding ability of the forkhead domain, which may serve as an auto-inhibitory feedback mechanism (22, 23).

Dysfunctional FOXP3 protein due to mutations in the *FOXP3* gene results in the development of severe autoimmune disorders as can be observed in the “scurfy” mouse mutant and patients suffering from immune dysregulation, polyendocrinopathy, enteropathy, and X-linked syndrome (IPEX) (24, 25). Most IPEX patients die within the first 2 years of life due to severe systemic autoimmune impairments. The most common FOXP3 mutations in IPEX patients are in the FKH domain, followed by the leucine zipper domain and the repressor domain (1, 24, 26). It was reported that the IPEX patient with mutated FOXP3FL but intact FOXP3ΔE2 protein, had mild autoimmunity and impaired Treg function, although the proportion of Tregs was shown even higher than that in healthy people (22).

### 2.2 Different functions of FOXP3FL and FOXP3ΔE2 isoforms

Earlier *in vitro* studies suggested that the FOXP3FL and FOXP3ΔE2 isoforms might show similar effects on mediating Treg differentiation and function, as ectopic expression of each isoform successfully induced Treg phenotypes from CD4^+^ T cells (27, 28). However, recent studies indicate that FOXP3 splicing isoforms show distinct abilities in the cofactor interaction, the nuclear translocation, and the DNA-binding ability, resulting in different effects on the differentiation, cytokine secretion, suppressive function, linage stability, and environmental adaptation of Tregs (12–15, 29–31).

FOXP3 regulates Tregs through complex mechanisms, including interacting with other transcription factors to form large transcription factor complexes (21). The loss of *FOXP3* exon 2 can not only impair the repressor domain function but also enhance the DNA-binding ability of the forkhead domain (22, 23), leading to different functions of FOXP3FL and FOXP3ΔE2 isoforms.

Retinoic acid receptor-related orphan receptor (ROR) α and RORγ are transcription factors belonging to the ROR family. They are expressed in many cell types including human CD4^+^ T cells and have an overlapping role in Th17-prone cell differentiation through the regulation of genes including IL17 and IL-22 (32, 33). Du et al. have shown that FOXP3 interacts with the activation function 2 (AF2) motif of RORα via an LxxLL motif (encoding aa 92-96 in the repressor domain) in exon 2 (**Figure 1**). FOXP3FL, but not FOXP3ΔE2, interacts with RORα and inhibits RORα-mediated transcriptional activation. Consequently, the mRNA expression of IL-17 and IL-22 is dramatically suppressed in Jurkat T cells expressing FOXP3FL (33).

Exon 2 of the *FOXP3* gene also includes a nuclear export sequence (NES) encoding aa 68-76 in the repressor domain of the FOXP3 protein (**Figure 1**). Magg et al. showed that site-directed mutagenesis of NES located in exon 2 completely abolished the nuclear export of FOXP3 in human T cells (30). Hence, the transport of FOXP3ΔE2 into the cytoplasm was much slower than FOXP3FL after cellular activation and it was more likely that FOXP3ΔE2 was trapped in the nucleus, resulting in increased expression of CD25 and C-C motif chemokine receptor 4 (CCR4), reduced expression of IL-2 and IL-4, and enhanced suppressive function of FOXP3ΔE2 *in vitro* (30). Yet it needs to be noted that, although the increased nuclear localization of FOXP3 is usually related to enhanced transcriptional activity, the interaction with cofactors in plasma may also be limited, possibly leading to more complex results in certain conditions.

Sato et al. used human CD4^+^ T cells in which the endogenous *FOXP3* gene was disrupted, followed by lentivirus-mediated transfer of FOXP3FL and FOXP3ΔE2 to dissect the role of each isoform and their combination (32) (**Table 1**). They found that co-expression of FOXP3FL and FOXP3ΔE2 induced higher FOXP3 protein expression when compared to the transfer of FOXP3FL alone or FOXP3ΔE2 alone (31) (**Table 1**). The transfer of FOXP3FL alone, FOXP3ΔE2 alone, and simultaneous expression of both isoforms led to comparable reductions of cytokines interleukin (IL)-2, IL-4, and Interferon-γ. In contrast, the co-expression of FOXP3FL and FOXP3ΔE2 caused a more pronounced reduction of IL-22 and IL-17A secretion compared to each isoform alone (31). Moreover, glycoprotein A repetitions predominant (GARP), which attaches the immunosuppressive cytokine transforming growth factor-beta (TGF-β) to the cell membrane, was mainly associated with the expression of the FOXP3ΔE2 isoform (31) (**Table 1**). Since these findings cannot be fully explained by the FOXP3 isoform-specific ROR interaction and nuclear translocation, there may be more complex mechanisms underlying the effects of FOXP3 isoforms on cytokine expression. An interaction between FOXP3FL and FOXP3ΔE2 may occur, contributing to the optimal FOXP3 protein expression and Treg phenotype. In addition, the different methods used in these studies may interfere with the comparative analysis. The supernatant cytokines detected by ELISA in the study of Sato et al. can differ from the intracellular mRNA expression in the studies of Du et al. and Magg et al. Post-transcriptional modifications should be taken into consideration.

**Table 1:** Effects of FOXP3FL, FOXP3ΔE2, and the co-expression of both isoforms on the total expression of FOXP3 and GARP and the secretion of critical cytokines in human *FOXP3*-knockout CD4^+^ T cells.

	Control	FL	ΔE2	FL/FL	ΔE2/ΔE2	FL/ΔE2
**FOXP3**	1.00	96.00	107.00	220.00	221.00	**300.00**
**GARP**	1.00	1.00	**2.50**	1.57	**5.11**	**5.25**
**IL-2**	1.00	0.14	0.07	0.15	0.14	0.11
**IL-4**	1.00	0.45	0.28	0.42	0.42	0.36
**INF-γ**	1.00	0.27	0.21	0.14	0.11	0.08
**IL-17A**	1.00	0.62	0.64	0.99	0.92	**0.57**
**IL-22**	1.00	0.80	0.75	0.51	0.58	**0.13**

We used available summary data which were published by Sato et al. (31). Sato et al. presented summary data of Treg-like characteristics of FOXP3-knockout CD4^+^ T cells transduced with FOXP3FL and/or FOXP3ΔE2 (31). These data included the protein expression of FOXP3, the mRNA expression of Glycoprotein A repetitions Predominant (GARP), and the supernatant concentrations of Interleukin (IL)-2, IL-4, Interferon (IFN)-γ, IL-17A, and IL-22. We calculated the ratios of these values in FOXP3-knockout cells with lentivirus-mediated single transduction of FOXP3FL(FL) or FOXP3ΔE2 (ΔE2), double transduction of FOXP3FL(FL/FL) or FOXP3ΔE2 (ΔE2/ΔE2), and co-transduction of both FOXP3FL and FOXP3ΔE2 (FL/ΔE2), with respect to the value in control FOXP3-knockout cells without FOXP3 transduction. The value in control FOXP3-knockout cells was set to 1. For example, in cells with double transduction of FOXP3FL, the expression of FOXP3 was 220 times, and in cells with co-transduction of FOXP3FL and FOXP3ΔE2, it was 300 times, compared to that in the control cells. Our calculation highlights the large magnitude of the effects produced by co-expression of FOXP3FL and FOXP3ΔE2. The most significant changes induced by the co-expression of FOXP3FL and FOXP3ΔE2 and the expression of FOXP3ΔE2 are highlighted in bold.

Recent studies strongly indicate that FOXP3 exon 2 controls Treg stability and autoimmunity. There are very few cells that exclusively express the FOXP3ΔE2 isoform in the intestine-resident CD4^+^ cells of healthy humans (29). In contrast, in various malignant tumor tissues, the expression of FOXP3ΔE2 far exceeds that of FOXP3FL (8) and FOXP3FL is even undetectable in some cancer cells (6, 34). It suggests that the FOXP3FL isoform may be mandatory for normal Tregs. Recently, Du et al. reported that deletion of *Foxp3* exon 2 in mice did not impact thymocyte development, but resulted in systemic autoimmune disease (12). Although FOXP3ΔE2 Tregs have comparable suppressive ability to FOXP3 FL *in vitro*, FOXP3ΔE2 Tregs *in vivo* exhibited intrinsic defects in the expression of phenotypic molecules including CD25, FOXP3, and Cytotoxic T-lymphocyte-associated protein 4 (CTLA-4). Purified FOXP3ΔE2 Tregs lost FOXP3 expression and were sufficient to induce systemic autoimmunity after being transduced to genetic immune-deficient mice (12). The crucial role of FOXP3FL in maintaining FOXP3 expression and mediating Treg stability and immune homeostasis is further elucidated with findings by Seitz et al. (14). They analyzed subsets of Tregs from two IPEX patients and a healthy carrier and found that FOXP3FL controlled a distinct genetic program, involving the identified FOXP3 regulators DNA-binding inhibitor 3 (*DI3*), B-cell lymphoma 6 (*BCL6*), and eukaryotic translation initiation factor 4E (*eIF4E*) (14). These FOXP3 regulators uphold Treg cell lineage stability, while they appear nonessential for Treg cell activation (35–37).

Moreover, FOXP3FL and FOXP3ΔE2 differ in the environmental adaptation of Tregs (22), which plays a fundamental role in modulating the local gut environment, aiding mucosal tolerance, and enforcing commensalism (38, 39). Tregs can be divided into two major subsets based on their expression of additional transcriptive factors. The first subset expresses RORγ induced by commensal bacteria, which is also a key regulator for Th17 cells (38, 40). The second subset expresses Helios, which is often considered a marker for natural Tregs generated from the thymus (38, 40). Recently, Gu et al. generated *Foxp3* exon 2-deficient mice by CRISPR-Cas9-based genome editing. They found that FOXP3ΔE2-bearing natural Tregs in the peripheral lymphoid organ were less sensitive to T cell antigen receptor (TCR) stimulation, due to the enhanced binding of FOXP3ΔE2 to the basic leucine zipper ATF-like transcription factor (BATF) promoter (22). In contrast, among peripherally induced RORγ^+^ Tregs in the colon, FOXP3ΔE2-expressing Tregs exhibited enhanced immune suppressive function over the wildtype FOXP3FL-expressing Tregs, due to the impaired FOXP3-RORγ interaction and the enhanced DNA-binding ability of the forkhead domain (22). It indicates that FOXP3ΔE2 has distinct effects on natural Tregs and peripherally induced Tregs and is beneficial for the adaptation of Tregs to the gut environment.

### 2.3 Changes of FOXP3FL and FOXP3ΔE2 isoforms in autoimmune and inflammatory diseases

Alternative splicing is a crucial post-transcriptional mechanism that enables reprogramming of gene expression profiles and the expansion of transcriptomic and proteomic diversity in eukaryotic organisms (41). Human *FOXP3* alternative splicing exists in both physiological and pathophysiological states (8, 42). FOXP3FL and FOXP3ΔE2 are usually co-expressed in healthy human lymphocytes, but their ratio may change according to the transcriptional environment (1). Several studies have investigated the changes of FOXP3 isoforms in human autoimmune diseases and inflammatory diseases with somewhat inconsistent findings (**Table 2**).

**Table 2:** Changes of FOXP3FL and FOXP3ΔE2 isoforms and their ratio in autoimmune diseases.

Disease (n)	Methods	Percentage in	ΔE2	FL	ΔE2/FL	FOXP3^+^ CD4^+/^CD4^+^ cells	Author (Reference)
**AAV** (43)	FC	CD4^+^ cells from PBMCs	↑ (ΔE2^+^ cells %)	↓ (FL^+^ cells %)	↑ (Ratio of cells)	↓	Free (39)
**HT** (10)	qPCR	PBMCs	↑ (ΔE2 RNA level)	— (FL RNA level)	↑ (Ratio of RNA)	?	Kristensen (43)
**Active GCA** (11)	FC	Ki67^+^ FOXP3^+^ CD4^+^ cells from PBMCs	↑ (ΔE2^+^ cells %)	↓ (FL^+^ cells %)	↑ (Ratio of cells)	?	Miyabe (44)
**RRMS** (13)	FC, qPCR	CD4^+^ CD25 ^high^ cells from PBMCs	↑ (ΔE2^+^ cells %)	↓ (FL^+^ cells %)	↑ (Ratio of cells)	?	Sambucci (45)
**CD** (20)	qPCR	CD4^+^ cells from small intestine	↑ (ΔE2 RNA level)	— (FL RNA level)	↑ (Ratio of RNA)	?	Serena (46)
**RA** (48)	PCR, FC	PBMCs	— (ΔE2 RNA level)	— (FL RNA level)	— (Ratio of RNA)	—	Lin (47)
**SLE** (50)	PCR, FC	PBMCs	— (ΔE2 RNA level)	— (FL RNA level)	— (Ratio of RNA)	↑	Lin (47)
**RA** (24)	qPCR	PBMCs	↓ (ΔE2 RNA level)	↓ (FL RNA level)	— (Ratio of RNA)	?	Suzuki (48)
**SLE** (26)	qPCR	PBMCs	↓ (ΔE2 RNA level)	↓ (FL RNA level)	— (Ratio of RNA)	?	Suzuki (48)
**IBD** (17)	qPCR, FC, IHC	mucosa (qPCR) or CD4^+^ cells from resected intestine	— (ΔE2 RNA level)	— (FL RNA level)	— (Ratio of RNA)	↑ (for Crohn’s Disease but not colitis)	Lord (29)
**MDS** (52)	FC	FOXP3^+^CD4^+^ cells	↓ (ΔE2^+^ cells %)	↑ (FL^+^ cells %)	↓ (Ratio of cells)	↓	Dudina (49)
**RRMS** (54)	WB, FC	CD4^+^CD25^+^CD127^−^ cells	↓ (44kDa band by WB)	↓ (47kDa band by WB)	?	— (By FC)	Carbone (50)

Compared to healthy control: ↑, increased; ↓, decreased; —, unchanged; ?, data unknown. n, number of patients enrolled; ΔE2, FOXP3ΔE2; FL, FOXP3FL. FOXP3^+^CD4^+^/CD4^+^ cells, the percentage of FOXP3^+^CD4^+^ cells within the CD4^+^ T-cell population; PBMCs, peripheral blood mononuclear cells; AAV, antineutrophil cytoplasmic antibody-associated vasculitis; HT, Hashimoto’s thyroiditis; GCA, giant cell arteritis; RRMS, relapsing-remitting multiple sclerosis; CD, coeliac disease; RA, rheumatoid arthritis; SLE, systemic lupus erythematosus; IBD, inflammatory bowel diseases; MDS, myelodysplastic syndromes; FC, flow cytometry; WB, Western blotting; PCR, reverse transcription-polymerase chain reaction; qPCR, quantitative PCR; IHC, immunohistochemistry.

Patients with antineutrophil cytoplasmic antibody-associated vasculitis (AAV) (39), Hashimoto’s thyroiditis (HT) (43), giant cell arteritis (GCA) (44), relapsing-remitting multiple sclerosis (RRMS) (45) and coeliac disease (CD) (46) showed upregulated FOXP3ΔE2 isoforms in peripheral blood mononuclear cells (PBMCs) compared to control subjects. On the other hand, patients with rheumatoid arthritis (RA) (47), systemic lupus erythematosus (SLE) (47, 48), inflammatory bowel diseases (IBD) (29), myelodysplastic syndromes (MDS) (49) and RRMS (50) showed lower or normal FOXP3ΔE2 isoform levels. The inconsistent results shown in the studies investigating the same disease might be partly due to the differences in the disease stages and the detection methods.

Joly et al. determined the impact of alternative splicing of *FOXP3* transcripts on atherosclerotic plaque stability in patients who underwent carotid endarterectomy (5). Real-time polymerase chain reaction (PCR) in a cohort of 150 patients indicated that higher plaque stability was associated with increased FOXP3ΔE2 isoform expression in the plaque (5). However, the FOXP3 isoform expression in peripheral blood mononuclear cells was not associated with plaque stability (5). Saleh et al. found that lower levels of total FOXP3 mRNA in PBMCs of kidney transplant recipients are associated with prolonged duration of inflammatory responses (51). Bruzzaniti et al. analyzed circulating peripheral Tregs in chronic obstructive pulmonary disease (COPD) subjects at different stages by staining cells with 2 specific FoxP3 antibodies: one that recognizes all splicing variants of FOXP3 and the other specific for FOXP3FL. Cytofluorimetric analysis revealed that both FOXP3FL and total FOXP3 frequencies increased at the early stage of COPD and decreased at the later exacerbated stage with severe inflammation (15). It indicated that the FOXP3FL isoform and the total FOXP3 level tend to correlate negatively with inflammation severity in disease progression.

There are some methodological concerns, about whether FOXP3 isoforms are causally associated with certain diseases: First, FOXP3 isoforms change during different stages of disease development, as shown in the studies on COPD and multiple sclerosis (7, 15, 45, 46). Second, FOXP3 splicing is organ-specific, which may result in differences between circulating T cells and tissue-resident T cells (5, 52). Third, studies using only PCR can’t show the changes in the percentage of FOXP3^+^ cells and the mean expression level per cell like the flow cytometry technique (12), especially when the total FOXP3 level remains unchanged in a group of CD4^+^ cells. Fourth, when flow cytometry is used to detect cell percentages, the difference in gating conditions may lead to distinct results, as is shown in Miyabe’s study (44). In this case, it’s better to analyze the absolute counting numbers of cell subsets rather than only percentages.

### 2.4 FOXP3 isoforms in cancer

Tregs are recruited to the tumor microenvironment and facilitate tumor cells to escape immune surveillance (53). Tumor-infiltrating Tregs can comprise up to 50% of intratumoral CD4+ T cells, exhibiting a more proliferative and immunosuppressive phenotype (54). High infiltration of Tregs correlates with poor prognosis in solid cancers including NSCLC, ovarian cancer, melanoma, and gastric cancer (53, 55). Thus, targeting tumor-infiltrating Tregs has become a promising anti-tumor strategy. Treg-targeting monoclonal antibodies against CD25, CTLA-4, Pd-1, and CCR4 are used to induce antitumor immunity (53). FOXP3 is a potential specific target for Treg suppression. FOXP3 in tumor-infiltrating CD4^+^ T cells is generally associated with an intrinsic capacity to suppress tumor immunity (53, 55). Therapeutic blocking of Tregs-specific FOXP3 reduced breast cancer growth in animal models (6). The prognostic value of CD4^+^FOXP3^+^ T cells has been confirmed in many cancers, but contradictory results have also been found in some cancers. Weed et al. demonstrated that, in patients with oral squamous cell carcinoma, the nuclear localization of FOXP3, rather than the overall expression of FOXP3, was associated with tumor recurrence within 3 years (56). As FOXP3 isoforms lacking exon 2 or exon 7 are more likely to be trapped in nuclei (30), the relationship between Treg FOXP3 isoforms and cancer prognosis needs further research.

Interestingly, FOXP3 is also expressed in various cancer cells and plays a complex role in tumor development. The functional roles of tumour-FOXP3 are inconsistent and even reversed. In breast cancer, gastric cancer, prostate cancer, and HCC, tumor FOXP3 acts as a tumor suppressor that inhibits the expression of multiple oncogenes. In contrast, tumor FOXP3 has been identified as a biomarker associated with malignant prognosis in pancreatic cancer, non-small cell lung cancer (NSCLC), thyroid cancer, and melanoma (34). The different roles of FOXP3 in cancer cells may be partly related to the alternative splicing which seems to produce more short isoforms than in normal cells. Jia et al. analyzed TCGA RNA-seq data from 9171 primary tumor tissues across 32 tumor types. Four FOXP3 isoforms including FOXP3FL, FOXP3ΔE2, FOXP3ΔE7, and an uncharacterized isoform were identified. FOXP3ΔE2 is dominant in most cancers except for acute myeloid leukemia (8). FOXP3ΔE2 over-expression in bladder cancer mediates cisplatin chemotherapy resistance (57). Besides, FOXP3ΔE2ΔE3, an isoform that doesn’t exist in Tregs, was identified in hepatocellular carcinoma cells and showed less inhibitory effect on tumor growth compared to FOXP3FL (58). By observing 10 malignant breast cancer cell lines, Zuo et al. found that none of the 10 cancer cell lines expressed FOXP3FL transcripts and 3 of them expressed FOXP3ΔE2ΔE3. The high incidence of somatic mutations contributed to the absence of FOXP3 or the abnormal expression of short FOXP3 splicing isoforms in breast cancer cells, leading to the impairment in HER2 oncogene suppression (59).

## 3 Metabolic regulation of FOXP3 transcription and splicing

### 3.1 Mechanisms controlling FOXP3 transcription and splicing during Tregs induction

**Figure 2:**
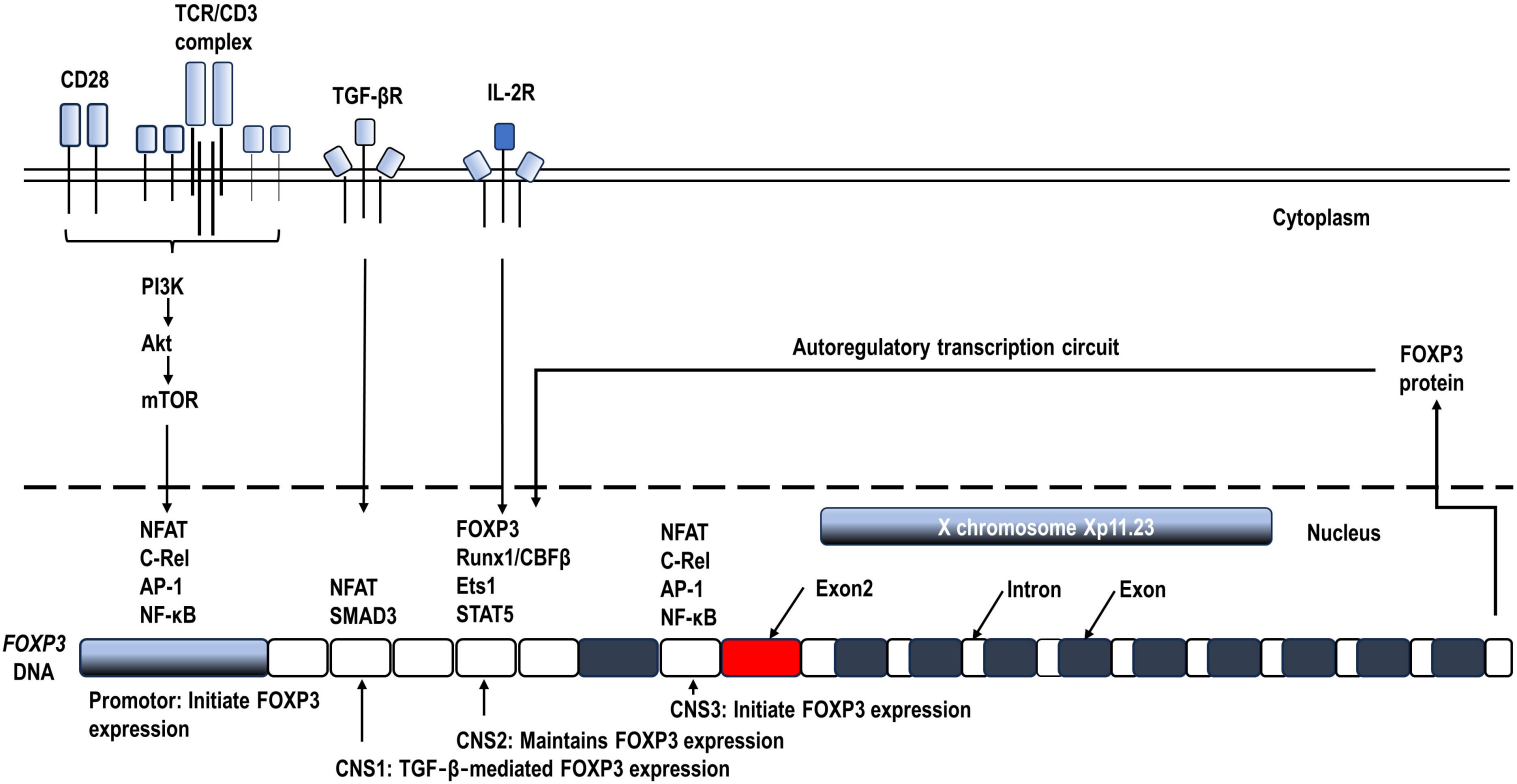
Schematic diagram of the mechanisms controlling *FOXP3* transcription. Continuous T cell antigen receptor (TCR) stimulation and CD28 co-stimulation, along with IL‐2 signaling are required for inducing and maintaining FOXP3 expression (60, 62, 63). The *FOXP3* gene locus contains 3 conserved non-coding sequences (CNS), which recruit transcription factor complexes to regulate FOXP3 transcription during Tregs activation and differentiation (1, 21).

Sustained FOXP3 expression, along with continuous TCR stimulation and CD28 co-stimulation, is necessary for Treg function (60, 61). In humans, anti-CD3/anti-CD28 co-stimulation of naïve CD4^+^ T cells can’t produce strong immunosuppressive capacity, although FOXP3 can be induced transiently (62). Signal transducer and activator of transcription 5 (STAT5) induced by IL‐2 signaling is required for maintaining FOXP3 expression (63). Furthermore, the TCR-driven Foxp3^+^ Tregs from naïve CD4^+^ T cells still require production and/or activation of TGF-β in serum-containing culture medium or in T cells (64). These essential signaling pathways are shown in **Figure 2**.

A series of key transcription factors have been identified to form large complexes binding to the conserved non-coding sequences (CNS) of *FOXP3* DNA and finely control *FOXP3* transcription (21, 65) (**Figure 2**). To initiate *FOXP3* transcription, transcription factors mainly including the nuclear factor of activated T cells (NFAT), nuclear factor kappa B (NF-κB), and activator protein 1 (AP-1) form an enhancing complex across the promotor and CNS3, integrating TCR and CD28 costimulatory signaling pathways in Treg precursors (66, 67). In addition, CNS1 enhances TCR‐ and TGF‐β‐induced *FOXP3* expression by binding NFAT and Smad3 (1, 21, 68). Prolonged CD28 signals can also inhibit *FOXP3* transcription by activating Akt/the mammalian target of rapamycin (mTOR) and thus inhibiting FOXO1 and FOXO3 transcription factors which are positive regulators of *FOXP3* expression (69). Once expressed, along with continuous TCR and CD28 co-stimulation, FOXP3 augments and stabilizes its own transcription by forming an autoregulatory transcription circuit, which likely involves the complex of FOXP3, Runx1/CBFβ, Ets1, and STAT5 binding to CNS2 (63, 70). The accessibility of CNS2 is ensured by the demethylation of its CpG islands, known as the Treg cell-specific demethylated region (TSDR), which serves as an indicator of the stability of FOXP3 expression and Treg phenotype (71, 72). Ozay et al. found that demethylated CpGs overlapped with the STAT5 binding site, consistent with a requirement for STAT5 binding for the maintenance of FOXP3 expression (73). These key transcription factors and CNS locus can additionally recruit other cofactors to further enhance or suppress FOXP3 transcription and splicing. The cofactors related to energy metabolism including glycolysis and oxidative phosphorylation will be reviewed in the next section.

The above mechanistic studies mainly focus on the regulation of the transcription process from FOXP3 DNA to pre-mRNA, which may indirectly affect the subsequent alternative splicing. Whereas, studies on the direct regulation of FOXP3 splicing are relatively rare. This posttranscriptional modification process relies on the identification of splice sites by ribonucleoproteins and the mature mRNA generation by spliceosomes. This step is regulated by exonic or intronic enhancer/silencer auxiliary elements (74). DNA methylation has been shown to influence alternative splicing decisions by either promoting exon inclusion via recruitment of the methyl-CpG-binding protein MeCP2 or promoting alternative exon skipping via increasing RNA polymerase II (RNAPII) elongation (75). Protein kinase C θ (PKCθ), a molecular regulator of TCR downstream signaling, has been shown to phosphorylate splicing factors and inhibit FOXP3 demethylation through its modulation of two key components of RNA processing, heterogeneous nuclear ribonucleoprotein L (hnRNPL) and protein-l-isoaspartate O-methyltransferase-1 (PCMT1) (73). Minato Hirano et al. showed that the splicing of FOXP3 is strongly dependent on the RNA helicase DExD/H-Box Polypeptide 39B (DDX39B) (76).

The dynamic changes of FOXP3 splicing isoforms have been identified during the ex vivo Tregs induction. Blinova et al. reported that anti-CD3/anti-CD28 co-stimulation combined with IL-2 and TGF-β produced high numbers of mature ex vivo Tregs over 7 days of cultivation from initial CD4^+^ T cells (7). The proportions of FOXP3 splicing isoforms demonstrated complex and dynamic expression profiles. FOXP3FL was repressed from about 40% of the total FOXP3 isoforms at the initial to about 10% after 3-5 days’ stimulation and significantly increased to more than 90% at days 7 and 9. FOXP3ΔE2 remained at about 40% of the total FOXP3 isoforms in the first 5 days and was detected at minor levels in mature Tregs on days 7 and 9. The expression of FOXP3ΔE7 increased up to 21.6% at days 3 and 5 but became almost undetectable in mature Tregs. A similar expression pattern was observed for FOXP3ΔE2ΔE7 (7). However, the molecular mechanisms underlying the distinct changes of FOXP3 isoforms need further research.

### 3.2 Metabolic regulation of FOXP3 transcription and splicing by glycolysis and fatty acid oxidation

Compared to T effector cells (Teffs), Tregs at a quiescent state express low surface levels of glucose transporter 1 (GLUT1), have more activated AMP-activated protein kinase (AMPK), are rich in mitochondria, and mainly depend on lipid oxidation (20, 77). This indicates that Tregs may mainly use fatty acid oxidation for energy production. This metabolic preference in Tregs is reported to be regulated by FOXP3, which inhibits the expression of GLUT1 (20), glycolytic enzymes, and the master regulator c-Myc (19). Inhibition of glucose uptake and oxidation in inflammatory mice models promotes Tregs rather than Teffs development both *in vitro* and *in vivo* (78). Deletion of *Glut1* in mice doesn’t affect the suppressive function of natural Tregs, or the *in vitro* Tregs induction (79). Genetically modified mice models also show that enhanced glycolysis in Tregs can decrease FOXP3 expression and disrupt their lineage stability and suppressive function (19, 80, 81). On the other hand, the treatment with etomoxir, a blocker of lipid oxidation through inhibition of carnitine palmitoyltransferase-1 (CPT1), decreases Tregs differentiation and function (77). Whereas elevated lipid oxidation by exogenous fatty acid addition or mTOR inhibitor rapamycin (82) results in modestly increased FOXP3 expression and Tregs differentiation (77, 83).

The above findings indicate that fatty acid oxidation is required for the differentiation and function maintenance of Tregs, while glycolysis is not necessary or even harmful to FOXP3 expression and functional Tregs differentiation in mice. However, it is becoming evident that this is not a fixed paradigm. Recent studies showed that activated Tregs in both mice and humans could engage in glycolytic metabolism ex vivo (84, 85). Kishore et al. showed that glycolysis initiated by the CD28-PI3K-mTORC2-mediated pathway led to glucokinase (GCK) induction, which interacted with actin filaments and promoted Tregs migration (86). Tanimine et al. observed that the glucose uptake inhibitor 2-deoxy-D-glucose (2-DG) at the onset of activation (the first 3 days) significantly decreased FOXP3 induction and cell proliferation, while at later stages of activation had little or no effect on either human thymically-derived or ex vivo-induced Tregs (87). These discoveries indicate that glycolysis is indispensable for Tregs in the initial activation, proliferation, and migration. Whereas, in the subsequent differentiation, Tregs exhibit more oxidative metabolism dependent on lipids and pyruvate and become less dependent on glucose (19, 87, 88).

**Figure 3:**
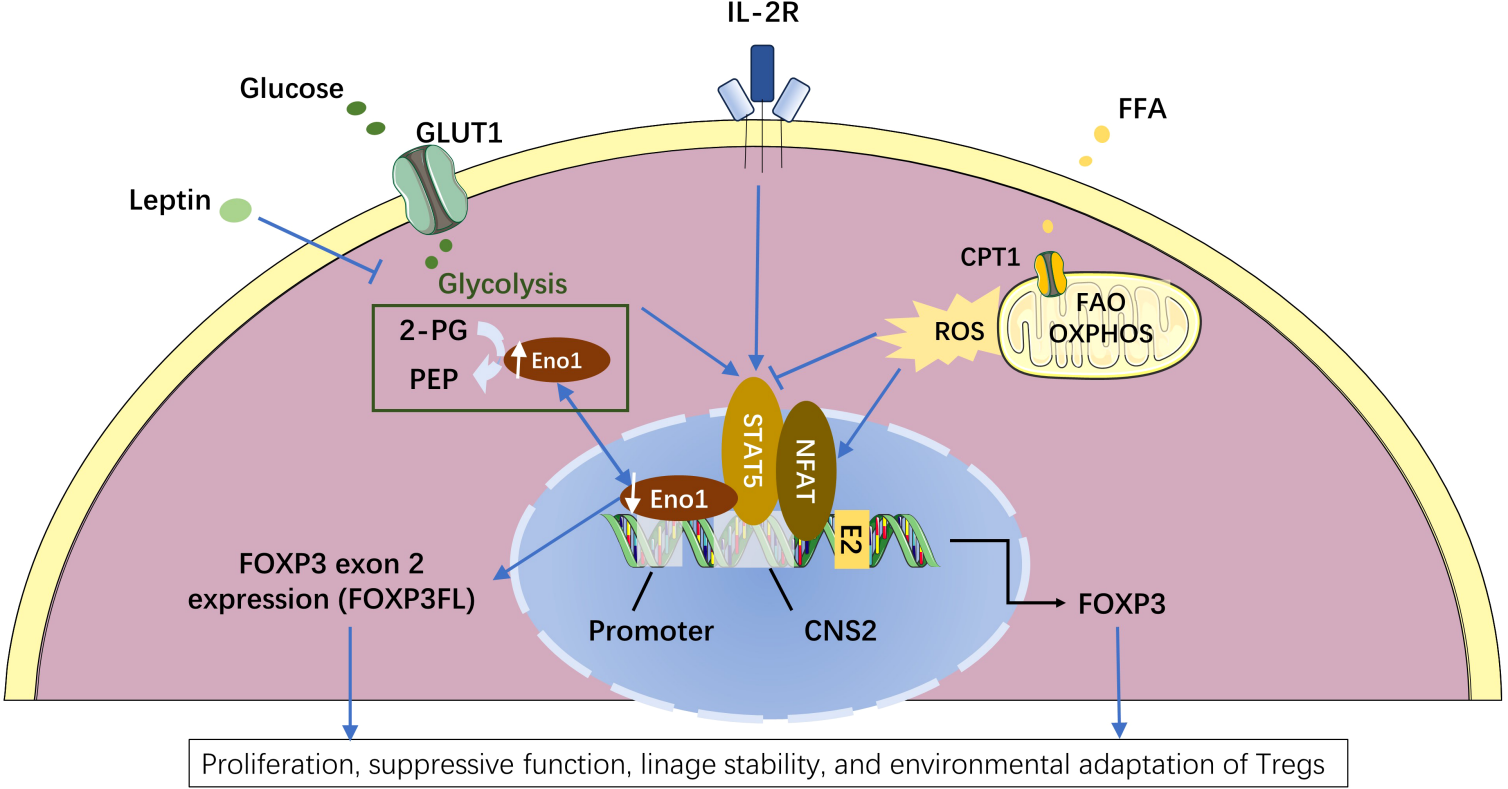
Metabolic regulation of FOXP3 splicing isoforms in the induced regulatory T cells (Tregs). Enolase-1 (Eno1), a glycolysis enzyme responsible for converting 2-phosphoglycerate (2-PG) to phosphoenolpyruvate (PEP), can also bind to the promoter and CNS2 locus of *FOXP3* DNA and hinder the exon 2 expression (16). Enhanced glycolysis leads to more Eno1 in the cytoplasm and less nuclear Eno1, and consequently reduced the binding of Eno1 to the promoter and CNS2 locus of *FOXP3*. The release of this Eno1-*FOXP3* binding specially promotes the expression of FOXP3 isoform containing exon 2 (FOXPFL) (16). While leptin can down-regulate FOXP3FL expression by inhibiting glycolysis (15). Compared to FOXP3 isoform lacking exon 2 (FOXP3ΔE2), FOXP3FL has stronger capacities in the proliferation, suppressive function, and lineage stability of Tregs (12, 16). Enhanced fatty acid oxidation (FAO) produces more mitochondrial reactive oxygen species (ROS) that can facilitate NFAT binding to the CNS2 enhancer of the *FOXP3* locus (81–83), leading to a slight increase in the total FOXP3 transcription (16). Besides, glycolysis can promote while fatty acid oxidation can inhibit IL2-induced STAT5 signaling which is critical for FOXP3 maintenance (16, 63). The Figure was partly generated using Servier Medical Art, provided by Servier, licensed under a Creative Commons Attribution 3.0 unported license.

Furthermore, research on FOXP3 isoform level indicates that glycolysis specifically promotes the expression of *FOXP3* exon 2 (16) which mainly contributes to the stability and the suppressive function of Tregs (12). De Rosa et al. found that glycolysis promoted FOXP3FL isoform expression through the glycolytic enzyme enolase-1, leading to enhanced induction and suppressive function of Tregs *in vitro* (16) (**Figure 3**). They induced CD4^+^CD25^+^ Tregs from human CD4^+^CD25^-^ cells by modest anti-CD3/anti-CD28 co-stimulation for 72 hours in the absence or presence of metabolic regulators during the first 36 hours. Tregs generated in the presence of 2-DG resulted in the nuclear translocation of enolase-1, which can bind to the promoter and CNS 2 regions of *FOXP3* DNA and hinder the exon 2 expression, leading to significant FOXP3FL reduction. Enolase-1 siRNA reverted the effects of 2-DG treatment (16). Whereas Tregs generated in the presence of the fatty acid oxidation inhibitor etomoxir demonstrated slightly higher expression of both FOXP3 splicing isoforms through enhancing IL-2/STAT5 signaling (16). The *FOXP3*-exon 2-related suppressive activity of Tregs was impaired in human autoimmune diseases, including multiple sclerosis and type 1 diabetes, and was associated with impaired glycolysis and signaling via IL-2 (16). This study reveals that glycolysis is required for the expression of FOXP3FL that contains exon 2 during the initial induction of Tregs from human CD4^+^CD25^-^ cells and unravels the underlying mechanism. However, this study’s inducing condition (weak TCR stimulation in the absence of TGF-β or IL-2) and duration (72 hours) differ from many other studies (7, 12, 87–89), which may not fully reflect the *in vivo* induction of Tregs. Despite the flaws, the necessity of glycolysis in *FOXP3* exon 2 expression and normal function of Tregs was confirmed in later studies on freshly isolated human Tregs. Bruzzaniti et al. found that leptin overproduction in patients with severe COPD inhibited glycolysis and FOXP3FL expression, and reduced the generation and function of Tregs isolated from the blood of COPD patients (15). Adriawan et al. identified the lower expression of glycolytic enzymes, such as phosphofructokinase and enolase 1, and downregulation of FOXP3 and CD25 as well as the reduced TCR-induced calcium influx as correlates of Tregs dysfunction in patients with Giant cell arteritis (GCA). They also observed that glycolysis inhibition in healthy Tregs led to higher frequencies of FOXP3ΔE2-expressing Tregs and inhibited CD25 upregulation after 18 hours of ex vivo TCR stimulation. Like ex vivo GCA Tregs, isolated FOXP3ΔE2-expressing Tregs expressed less CD25 than FOXP3FL-expressing Tregs (90).

Therefore, it’s clear that glycolysis and fatty acid oxidation have respectively different effects on the expression of FOXP3 splicing isoforms and in the different activation stages of Tregs. Procaccini et al. illustrated a proteomic and metabolic comparison between human Tregs and CD4^+^CD25^-^ conventional T (Tconv) cells in both freshly isolated and *in vitro* culture conditions. They found that freshly isolated human Tregs were highly glycolytic as compared to Tconv cells. The *in vitro* proliferation, FOXP3 expression, and suppressive activity of Tregs, induced by leptin neutralization, required both glycolysis and fatty acid oxidation, whereas those of Tconv cells require only glycolysis (85). Zhu et al. used liquid chromatography and tandem mass spectrometry (LC-MS/MS) to analyze the metabolic profiles of freshly isolated spleen Tregs in mice with sepsis with or without treatment with 2-DG or etomoxir. It was found that in severe infection, activated Tregs depend on glycolysis and fatty acid oxidation, and inhibition of metabolic pathways by either 2-DG or etomoxir reduced FOXP3 expression and increased Treg apoptosis (91). These studies confirm that both glycolysis and fatty acid oxidation are required *in vivo* for activated functional Tregs in both mice and humans (85, 91, 92). It may be helpful to further clarify the respective percentages of glycolysis and fatty acid oxidation in the total energy production in Tregs at different activation stages.

### 3.3 Metabolic regulation of FOXP3 transcription by mitochondria and reactive oxygen species

Both natural and induced Tregs have high mitochondrial mass and excessive reactive oxygen species (ROS) production (93). In activated conditions, Tregs have continuously higher ROS production compared to Teffs (19). ROS are highly reactive byproducts of normal oxygen metabolism and are involved in cellular signal transduction (94, 95), playing critical roles in human chronic inflammatory diseases (96, 97). ROS from mitochondrial oxidative phosphorylation (OXPHOS) activates NFAT in the nucleus, which then binds to the CNS2 enhancer of the *FOXP3* gene and stimulates its expression (98–100) (**Figure 3**). In turn, FOXP3 can alter T cell metabolism and increase OXPHOS to adapt to the environment (19, 101). It has been demonstrated that Tregs exhibit reduced sensitivity to oxidative stress-induced cell death and maintain their suppressive function in a high ROS environment (102). These studies indicate that ROS generated from OXPHOS can enhance FOXP3 expression and promote the functional survival of activated Tregs.

However, excessive ROS can serve as a key mediator for promoting Treg instability in autoimmune diseases like antineutrophil cytoplasmic antibody-associated vasculitis (103) and impairing Tregs/Teffs homeostasis in cardiovascular diseases (104, 105). NADPH oxidase 2 (Nox2) is a ROS-generating enzyme that has been identified in CD4^+^CD25^+^FoxP3^+^ Tregs (104). Nox2-deficient Tregs produced less ROS, had increased nuclear levels of FOXP3 and NF-κB activation, and were more suppressive than wildtype Tregs (104). Compared to the wildtype, mice with CD4-targeted Nox2 deficiency had significantly higher infiltration of Tregs in the heart and minor Angiotensin II-dependent cardiovascular damage (104).

Besides ROS generation, mitochondria play a critical role in the switch of energy substances. Enhanced mitochondrial function benefits fatty acid oxidation and aerobic metabolism (77, 106). The mitochondrial transcription factor A (Tfam), which is essential for mitochondrial respiration, is also indispensable for the maintenance of Treg suppressive capacity. The genetic deletion of Tfam in Tregs impaired both their proliferation and function by enhancing DNA methylation in the TSDR of the *FOXP3* locus (107).

### 3.4 Critical metabolic signaling pathways in the regulation of FOXP3 expression

Various signaling pathways are involved in the immunometabolism regulation of Tregs. PI3K/mTOR signaling serves as a critical link between the activating stimuli and the metabolic regulators in Tregs. Signals through the TCR, CD28, or IL-2 receptor activate the PI3K/Akt/mTOR cascade (108), which is critical for FOXP3 expression (109). mTOR activity is finely tuned by various metabolic signals (110). In response to ATP depletion, AMPK activation (111) promotes fatty acid oxidation while inhibiting mTOR-mediated glycolysis (77). The hypoxia-inducible transcription factor 1α (HIF-1α), a vital element in adaptation to varying oxygen states (112), is also activated in response to TCR activation and mTOR-mediated glycolysis (113–115). Short-chain fatty acids (SCFA) in the gut are also known to inhibit mTOR (116). Butyrate, one type of SCFA generated from gut microbiota, is reported to regulate the balance between FOXP3 isoforms in human gut-resident Tregs (46), but whether this effect of butyrate is mediated by the mTOR signaling pathway needs further verification. Besides, dietary nutrients and specific gut microbiota can also activate another transcriptional factor, aryl hydrocarbon receptor (AhR), which promotes FOXP3 expression and Treg development (117–119). Yu Y et al. revealed that the dietary intake of a moderate dose of glucose (6% w/v) induced Tregs in guts and alleviated colitis development in mice, and the mechanism might be related to glucose-induced AhR activation (119). Generally, the above-mentioned metabolic signaling pathways are closely related to both glycolysis and FOXP3 expression, but their specific roles in regulating FOXP3 alternative splicing need further research.

## 4 Concluding remarks and future perspectives

FOXP3 is crucial for the functional maintenance of human Tregs. The predominant FOXP3 splicing isoforms, FOXP3FL and FOXP3ΔE2, differ in functions and metabolic regulation mechanisms. The balance of FOXP3 splicing isoforms is related to autoimmune diseases, inflammatory diseases, and cancers. In response to environmental challenges, FOXP3 transcription and splicing can be finely regulated by TCR stimulation, glycolysis, fatty acid oxidation, and mitochondrial capacity. Targeting energy metabolism and FOXP3 splicing isoforms may provide new therapeutic approaches for Treg-related diseases.

Modulating Treg function has emerged as a promising approach to either upregulate or downregulate suppressive activity in the context of autoimmunity or cancer, respectively. Ex vivo expanded antigen-specific Tregs may be injected into patients with autoimmune diseases to restore Treg function (120). The stability of ex vivo expanded Tregs is one of the limitations associated with Tregs therapy (121). Proper approaches affecting glycolysis may help to balance FOXP3 isoforms, promote FOXP3 stability, and optimize Treg quality and quantity. For anti-cancer therapy, FOXP3 is a potential new target for specific Treg suppression; however, the nuclear FOXP3 is considered inaccessible by traditional therapeutics. The next-generation antisense oligonucleotides and TCR mimic antibodies targeting FOXP3-derived peptides have been developed to improve the nuclear FOXP3 accessibility in mouse models (53). Therapeutics using small molecules and splice-switching oligonucleotides may modulate alternative RNA splicing process and generate cancer-specific transcripts leading to altered functions (122, 123). However, currently no therapeutics have been described for the alternative splicing of FOXP3. Furthermore, *in vivo* application of metabolic regulating medicines can lead to complex effects because many types of cells can be involved. Thus, it is still a great challenge to specifically target the critical metabolic enzymes related to FOXP3 isoforms in Tregs.

The field of FOXP3 splicing isoform research is still emerging. Further studies would fully elucidate the roles of FOXP3 splicing isoforms in the development of diseases and the metabolic mechanisms regulating FOXP3 splicing. As regulation of FOXP3 expression may occur at various levels including transcriptional, RNA splicing, and post-transcriptional levels (71), methods like flow cytometry, Western blot, and immunohistochemistry using isoform-specific antibodies in combination with quantitative PCR may be required to fully demonstrate the changes in FOXP3 expression and distribution. Furthermore, Tregs from different tissues or at different disease stages, and as well as the different ex vivo treatment procedures of Tregs, may produce distinct results in FOXP3 expression and Treg function, and thus, these methods should be properly addressed and compared.

## Author contributions

ZL: Funding acquisition, Writing – original draft, Writing – review & editing. YZ: Writing – original draft, Writing – review & editing. QS: Writing – review & editing. JZ: Writing – review & editing. ZZ: Funding acquisition, Writing – review & editing. MT: Conceptualization, Investigation, Project administration, Supervision, Writing – review & editing.
